# The 2014–2015 Ebola outbreak in West Africa: Hands On

**DOI:** 10.1186/s13756-016-0112-9

**Published:** 2016-05-05

**Authors:** Pauline Vetter, Julie-Anne Dayer, Manuel Schibler, Benedetta Allegranzi, Donal Brown, Alexandra Calmy, Derek Christie, Sergey Eremin, Olivier Hagon, David Henderson, Anne Iten, Edward Kelley, Frederick Marais, Babacar Ndoye, Jérôme Pugin, Hugues Robert-Nicoud, Esther Sterk, Michael Tapper, Claire-Anne Siegrist, Laurent Kaiser, Didier Pittet

**Affiliations:** Infection Control Programme and WHO Collaborating Centre on Patient Safety, University Hospitals and University of Geneva Medical School, Geneva, Switzerland; Division of Infectious Diseases, Laboratory of Virology and Swiss Reference Centre for Emerging Viral Diseases, University of Geneva Hospitals, Geneva, Switzerland; World Health Organization, Geneva, Switzerland; UK Department for International Development, London, UK; HIV unit, Division of Infectious Diseases, University of Geneva Hospitals, Geneva, Switzerland; Institute of Global Health, University of Geneva, Geneva, Switzerland; Laboratory of Urban Sociology, EPFL, Lausanne, Switzerland; Division of Tropical and Humanitarian Medicine, Geneva University Hospitals, Geneva, Switzerland; National Institutes of Health (NIH) Clinical Center, Washington DC, USA; Western Cape Government and Stellenbosch University, Cape Town, South Africa; Expert-consultant and trainer in hospital hygiene, infection control and patient safety for WHO and UNDP, Dakar, Senegal; Intensive Care Unit, University of Geneva Hospitals, Geneva, Switzerland; Médecins sans Frontières, Geneva Operational Centre, Geneva, Switzerland; Division of Infectious Diseases, Lenox Hill Hospital, New York, USA; Departments of Pathology, Immunology and Paediatrics, WHO Collaborating Centre for Vaccine Immunology, University of Geneva Hospitals, Geneva, Switzerland

## Abstract

The International Consortium for Prevention and Infection Control (ICPIC) organises a biannual conference (ICPIC) on various subjects related to infection prevention, treatment and control. During ICPIC 2015, held in Geneva in June 2015, a full one-day session focused on the 2014–2015 Ebola virus disease (EVD) outbreak in West Africa. This article is a non-exhaustive compilation of these discussions. It concentrates on lessons learned and imagining a way forward for the communities most affected by the epidemic. The reader can access video recordings of all lectures delivered during this one-day session, as referenced. Topics include the timeline of the international response, linkages between the dynamics of the epidemic and infection prevention and control, the importance of community engagement, and updates on virology, diagnosis, treatment and vaccination issues. The paper also includes discussions from public health, infectious diseases, critical care and infection control experts who cared for patients with EVD in Africa, in Europe, and in the United Sates and were involved in Ebola preparedness in both high- and low-resource settings and countries. This review concludes that too little is known about the pathogenesis and treatment of EVD, therefore basic and applied research in this area are urgently required. Furthermore, it is clear that epidemic preparedness needs to improve globally, in particular through the strengthening of health systems at local and national levels. There is a strong need for culturally sensitive approaches to public health which could be designed and delivered by social scientists and medical professionals working together. As of December 2015, this epidemic killed more than 11,000 people and infected more than 28,000; it has also generated more than 17,000 survivors and orphans, many of whom face somatic and psychological complications. The continued treatment and rehabilitation of these people is a public health priority, which also requires an integration of specific medical and social science approaches, not always available in West Africa.

## The Ebola virus

The emergence of Ebola viruses should be considered under the dual perspective of a large field of emerging viruses and considerable complexity and diversity among Ebola viruses themselves. Between 50 and 60 viruses are known to infect humans. Around 80 % are shared with animals. Unexpected viral outbreaks affecting humans over the past century include Yellow fever, Chikungunya, Dengue, West Nile Virus, MERS-CoV, Enterovirus 68, Enterovirus 71, Zikavirus, Japanese encephalitis, Hantavirus, Lassa, Marburg, Rift Valley, Crimean-Congo fever, Nipah and Hendra.

Compared to other viruses, Ebola virus is large and long – almost visible in an optical microscope – but has a small genome coding for 7 genes [1]. It is a RNA virus, meaning that it must continually replicate or die. Whereas DNA viruses or retroviruses have strategies such as latency, integration, chronic infection or reactivation, this is not an option for RNA viruses and explains why they often produce short and acute infections.

The Ebola virus genome is a 19 kB single RNA strand around 1000 nm in length [1]. As with other RNA viruses, the mutation rate is high: around one error per 10,000 to 100 000 nucleotides, which corresponds to one mistake per replication on average. Furthermore, gene exchange and recombination between viruses is possible. On the whole, the Ebola virus displays a high diversity, a high mutation rate, and it also probably has a large animal reservoir. These are three contributing factors that make epidemics probable.

Species barriers are important factors when considering viruses. For example, smallpox in humans is related to mousepox or camelpox, but each can infect only a single species due to genetic differences that are in the range of only 1–2 % [2]. In the case of the Ebola virus, fruit bats are a likely reservoir, but, antilope, rodents, and perhaps other mammals may also play a role [1]. The virus can also be transmitted to humans from apes, which are accidental hosts. Overall, the prevalence of Ebola virus disease (EVD) infections in non-human primates is not known and more research is clearly needed in this area.

In the case of the 2014–2015 EVD outbreak in West Africa, RT-PCR analysis and sequencing have shown that the pathogen is a Zaire Ebola virus. The variant involved in this outbreak has been named Makona, by the name of a river running through the area between Guinea, Liberia and Sierra Leone where the outbreak was first declared.

Pending further investigations, the outbreak was probably a zoonotic event, which was transmitted to humans via an index case in Guéckédou district, Guinea; [3] then human to human transmission ensued. Genetic analysis shows that the Makona variant emerged from a common ancestor in 2004 [4]. The mutation rate of the Makona variant is in the expected range [5, 6]. However, it is not yet known in detail what the similarities and differences are between the 1976 and 1995 Kikwit variants and the current Makona variant. Recent research shows that this variant is constantly evolving but not fundamentally changing [7]. However, the fact that the virus sequences were not freely available online constituted a failure of the global response to this epidemic.

It should be remembered that another EVD outbreak occurred recently, in Democratic Republic of Congo, in July 2014 [8]. It affected Boende town in Equateur province, where there were 69 cases and 49 deaths, implying a 74 % fatality rate. Fortunately, the epidemic was rapidly brought under control. Analyses have shown that the virus was also a variant of the Zaire Ebolavirus species, with 96.8 % genetic identity to the current Makona variant.

Viral load at diagnosis is clearly linked to survival [9–12]. There are indications that the viral load in the present Makona epidemic may be up to 1000 times higher than in the Boende/Kikwit epidemic [13]. Moreover, clinical course in cynomologus macaques infected with the Makona variant seems to be slightly different compared to what has previously been observed with other variants; death occurs later in the course of the disease, and diarrhoea is more profuse, increasing the spread of infectious body fluids [14]. Furthermore, while no difference has been shown in decay rates of the Makona variant compared to the historical Yambuku variant in different matrices, the former seems to be more resistant during the drying process in human blood in experimental conditions mimicking the West African environment [15]. Those specific viral factors, combined with geographic, economic, social, and cultural determinants might explain the rapid expansion of the EVD epidemic in West Africa. Indeed, this outbreak marked the first reported EVD in this part of Africa; the lack of preparedness in countries suffering from poor health infrastructure, the geographic situation at the border of 3 different countries near major road networks enabling important human mobility to major capital cities, rooted beliefs in traditional medicine, burial practices, and reluctance to and fear of official health interventions have all participated to the unprecedented spread of the virus [16]. As in many other viral outbreaks, super spreaders are a significant problem [17]. For example, in 2014, a burial ceremony in Kenema, Sierra Leone, gave rise to 345 secondary cases [18].

It should be made clear that future outbreaks of EVD cannot be predicted and that much is still unknown about the biology and pathogenesis of the virus. For example, the receptors on human cell surfaces to which the Ebola virus attaches are not well known and are a priority for future research.

## Timeline of the response

Video at https://www.youtube.com/watch?v=hFK-Bzy5c6M and https://www.youtube.com/watch?v=TrBQM-C9CB8

Between 2000 and 2015, progress in international collaboration on issues related to infectious threats often occurred in the wake of epidemics or crises. The creation of the *Global Alliance for Vaccines and Immunization* (GAVI) and of the *Global Outbreak Alert and Response Network (*GOARN) in 2000, the advent of the International Health Regulations (IHR) in 2005 and the Pandemic Influenza Framework Preparedness (PIP) in 2011 paved the way for improvements in the international management of such threats. Over the same time frame, several serious local, regional or global outbreaks occurred, including SARS, H5N1, H1N1, cholera, MERS-CoV, H7N9 and Ebola.

The 2014–2015 EVD epidemic is exceptional in its magnitude, speed, severity, and international spread over six countries in West Africa; as well as in Guinea, Liberia and Sierra Leone, cases were recorded in Nigeria, Senegal and Mali [19]. The global response against it is also without precedent, bridging together many national and international partners and the first time ever United Nations emergency health mission: UNMEER (United Nations mission for Ebola emergency response).

The World Health Organization set up a roadmap from 28 August 2014 with three main objectives (Table 1) [20]. Activities to achieve these objectives covered case management, case diagnosis, surveillance, safe burials and social mobilisation. To limit the spread of the epidemic, extraordinary measures were implemented as provided by the International Health Regulations (HIR) framework: mass gatherings were deferred and there were temporary recommendations for limiting travel.

**Table 1:** Ebola - Major objectives of the World Health Organization roadmap (28 August 2014)

- Achieve full coverage with an Ebola intervention package in countries with widespread transmission	
- Ensure rapid and comprehensive interventions in countries with an initial case or localised transmission	
- Strengthen preparedness of all countries – especially those in close contact with areas with intense transmission	

As of June 2015, the international response coordinated by WHO and its partners involved over 2000 foreign medical staff belonging to 58 medical teams from 40 organisations, who provided support to 66 Ebola treatment centres (ETC) and more than 800 community hospitals and centres. Over 4000 health care workers (HCWs) were trained in affected countries and 1.5 million personal protective equipment (PPE) sets were distributed. Disease detection was enhanced by the contributions of 900 epidemiologists and 23 WHO Collaborating Centres belonging to the Emerging and Dangerous Pathogens Laboratory Network (EDPLN). Ebola preparedness plans were implemented in 15 countries: 45 guidance documents were published by WHO and its partners between March 2014 and June 2015, on topics ranging from infection prevention to safe burials.

In retrospect, three phases can be observed in this epidemic. At first, the response was based on available capacities at local and country levels, then a massive scaling up occurred with international support. The third phase relies much more on community engagement, which is essential in at least three areas: service delivery (early diagnosis, safe care, safe and dignified burials), planning and implementing services, and service uptake (advocacy, education and information, monitoring). The priority here is to ensure essential services and lay the foundation for health sector recovery and the strengthening of national core capacities.

The bridge between emergency response and health systems recovery and strengthening is critical because of the impact that the EVD epidemic has had on health systems in the three most affected countries. In Liberia, there was a 23 % decrease in institutional childbirths, a 39 % decrease in children treated for malaria, a 21 % decrease in childhood immunisation and as much as a 90 % drop in family planning visits. The recovery and development of health systems implies short-term capacity building in the health sector as well as a shift towards a community approach, which is one of the most salient elements of the WHO roadmap. Assigning a family member as the interface between family and health centre has proven to be an efficacious way of involving families and the community at large, and constitutes a best practice.

## Médecins sans Frontières

Video at https://www.youtube.com/watch?v=el0zhxCCgnQ

Médecins sans Frontières (MSF) played an essential role throughout the EVD outbreak and especially in its initial stages. This organization had experience from having been active in almost all filovirus outbreaks in Africa since 1995. From 2011, MSF had also been running a malaria programme in Guéckédou, Guinea, where the index case of the 2014–2015 EVD epidemic was identified. In March 2014, the Ministry of Health of Guinea transmitted to MSF 15 case descriptions, of which nine had been fatal. All were from the same family group or were staff from the local hospital. The report was transmitted to MSF headquarters in Geneva where Ebola virus infection was rapidly suspected because one of the cases was described as having hiccups. MSF raised the alarm within 48 h and sent further staff to the field in the following days. The affected population was mobile and crossing borders, so the very first step was to isolate patients. This rapidly evolved into a six-pillar Ebola management system (Table 2).

**Table 2:** The 6 pillars of Ebola management system, MSF

- isolation	
- outreach	
- safe burials	
- health promotion	
- psycho-social support	
- contact tracing	

On week 25 following the identification of the index case, MSF declared the epidemic out of control. On week 33, MSF took over the management of a large ETC in Foya, Northern Liberia, where two HCWs had been infected. The progression of the epidemic led MSF to set up the largest ETC, in Monrovia, with 240 beds. Altogether, MSF has been running 15 medical centres and two survivor clinics (in Freetown and Monrovia) during the epidemic. As of June 2015, some 6500 staff members have been involved, caring for 8509 patients among which 5177 were confirmed as EVD cases and 2449 survived (47.3 % survival rate). A total of 28 MSF staff became infected with Ebola virus (three were expatriates from Europe or North America) and 14 (50 %) survived.

The outbreak response in the two largest cities in the region, Freetown and Monrovia, was qualitatively and quantitatively different from work in rural areas: it implied larger medical centres, survivor clinics, and measures to avoid malaria patients going to hospitals, such as the distribution of antimalarial drugs at community level. In parallel, knowledge transfer was taking place, for staff within MSF and for other organisations involved in the response.

**Fig. 1:**
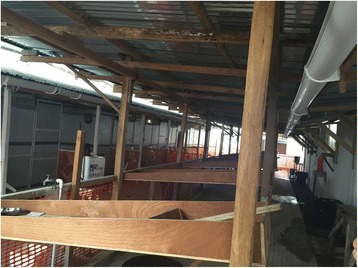
Ebola treatment centre (ETC), Redemption Hospital, Monrovia, Liberia. Man-made, inexpensive structures allowing for parents and relatives to visit of Ebola virus disease patients and bring them food and other personal items without risking close contact. The wooden structures allow the maintenance of a critical distance between EVD patient and visitors to be respected (photo, D. Pittet)

Looking at lessons learned, it should be considered that this outbreak had more cases than the total of all previous EVD outbreaks. MSF had never had to manage more than 40 beds or two medical centres before, had never been confronted by a new disease crossing borders, and had never had staff infected. The first lesson is that qualified HCWs continued to volunteer with MSF throughout the crisis; there was no recruitment problem. The second lesson is that some specific places are particularly dangerous, such as triage points, which are dangerous for staff where many of the infections probably occurred. Triage areas and suspect areas tend to group together people with symptoms that could be either EVD or malaria. It is therefore vitally important to supply individual rooms, with low risk corridors leading through high-risk areas. Structures allowing EVD patient to be visited with no risk for cross-transmission are also critical (see Fig. 1). The ETC in Foya, managed by MSF, with safe corridors leading through the high-risk zone, was the only setting where the outbreak was brought under control within three months. With hindsight, most actors in the field would agree that the international reaction to the EVD outbreak was too slow. Although this requires further research, the existence of a treatment might have accelerated the response. In any case, another lesson learned is that, facing epidemics with high transmission and fatality rates, experimental treatments need to be made available very quickly.

Finally, the importance of basic infection prevention and control (IPC) measures cannot be overstated. The majority of primary and secondary care facilities in the three most affected countries did not have basic IPC in place when the epidemic began. This clearly facilitated Ebola virus transmission. MSF staff in the field observed that basic IPC measures were still lacking in August-September 2015 in several clinics in rural areas. IPC should therefore be pinpointed as a key area for international investment and concern.

## The response in Sierra Leone

Video at https://www.youtube.com/watch?v=jX4xpgMbCiU

Under the umbrella of the United Nations and with the full agreement and cooperation of the national governments involved, it can be said that France (as well as French NGOs) played an important role in the Ebola response in Guinea, while the USA played an important role in Liberia and the United Kingdom (UK) in Sierra Leone.

Almost exactly two months after the declaration of the EVD outbreak in Guinea, on 22 March 2014, Sierra Leone confirmed that it was also affected, on 24 May 2014. The UK set up a Joint Inter-Agency Task Force (JIATF) led by the UK Department for International Development, including the Ministry of Defence, and Foreign and Commonwealth Office, to provide the planning, infrastructure, training and management required to scale up the response. Work was carried out in close collaboration with the Government of Sierra Leone to ensure coordination at national and district level, and support for the UN to tackle systemic issues.

An initial objective was to increase the number of treatment beds, which meant creating physical infrastructure using local contractors and military oversight, while ensuring adequate supplies and making sure that staff would be available to deliver treatment. Community Care Centres (with safe isolation beds) were set up, where individuals could present with early symptoms for early isolation, testing and referral. Laboratories were created to speed up diagnosis of EVD. IPC measures, social mobilisation, contact tracing and safe burial services were implemented to reduce the number of people needing treatment. Support was provided for robust contingency planning and regional preparedness to avoid spread to the wider region.

In September 2014, around 500 new cases were being declared every week in Sierra Leone alone, and some scenarios foresaw an exponential spread. In the field, the perception was that the international community had not moved early enough, and that the response was too slow. Nevertheless, on 19 November 2014, a JIATF was set up under UK coordination, with contributions from Canada, Norway and South Korea. The task force included civilian and military personnel, under civilian control.

When the task force arrived in Freetown, most bodies of EVD victims were not buried. The army of Sierra Leone responded efficiently to the challenge of ensuring safe and dignified burials, with the support of the task force. More generally, one of the lessons learned from the EVD epidemic is that there is an advantage in creating a rapid civilian-military response. Another lesson is that humanitarian, healthcare and even military sectors need to learn to work together, especially at district and community level – because this is where struggles against threats such as Ebola are lost or won.

## Linkages between the Ebola crisis and infection control

Video at https://www.youtube.com/watch?v=BTVysm-et00

The 2014–2015 EVD epidemic was complex because it transited rapidly between rural and urban areas and across borders, in a post-war environment marked by poverty and low literacy levels. The three most affected countries were lacking in infrastructure for transport, clean water, sanitation and public health. Furthermore, certain cultural practices and customs (especially around burials) were likely to fuel transmission. This situation was compounded by poor adherence to International Health Regulations and a belated response by the international community.

Specifically, the absence of basic IPC measures can be identified as a key area that led the epidemic to become so complex and widespread. In the three most involved countries, the situation before the epidemic began was already unsatisfactory, in the community as well as in healthcare settings.

In March 2015, WHO published an investigation of over 66,000 healthcare facilities in 54 low and middle-income countries, showing that 38 % had no clean water source, 19 % had no improved sanitation, and 35 % lacked access to soap and water for handwashing [21]. In the WHO AFRO Region as a whole, only 58 % of healthcare facilities had a controlled water supply in a 500 m radius, 84 % had adequate sanitation and 64 % had access to soap. Figures for the three countries most affected by the EVD epidemic are probably in the same range, however many of the statistics are either outdated or unavailable. The most important fact is that it is practically impossible to set up infection control measures without access to clean water, that “adequate sanitation” often amounts to a single toilet for an entire hospital, and that even access to soap remains a significant challenge for many hospitals.

A confidential assessment of over 100 public and private healthcare centres was carried out in February 2015. Results were not necessarily applicable to the whole of the three affected countries; however, WHO found that several key indicators were lacking. Over half of the surveyed facilities did not had secured access to clean water or electricity, nor did they had a functioning incinerator. Less than one quarter of the centres had isolation facilities and only half had triage facilities. Waste management was also found to be lacking in most centres. Although some positive elements were noted, such as adequate training of healthcare staff and good injection safety procedures, the situation as a whole was clearly unsatisfactory.

Another key lesson learned concerns communication, which becomes difficult when key actors come under the dual influence of fear and time constraints. In such situations, it is of paramount importance to adhere to standards and to remain evidence-based when editing the numerous guidance documents that must be published during the course of any major outbreak. During the course of the 2014–2015 EVD epidemic, there was a media obsession for PPE, while other very important measures such as hand hygiene, clean water or functioning toilets were rarely mentioned. Because PPE is an important measure for EVD control, WHO issued rapid guidance on the subject during the course of the epidemic, based on expert consultations [22]. Using a similar rapid consultation process, WHO issued interim guidance at the end of 2014 suggesting that routine spraying using chlorine solutions should not be recommended.

Infection rates among HCWs were intolerably high during the course of the EVD outbreak, with levels up to 42 times higher than for the general population [23–25]. Lack of triage and isolation facilities have been pinpointed as key areas for improvement, as well as previously mentioned IPC measures. Finally, the EVD outbreak, however tragic, may be viewed as an opportunity for improving hygiene in healthcare and community settings, using culturally sensitive methods based on social mobilisation and partner coordination at local, national and international levels.

## Practical infection control field intervention in Liberia and Guinea

Video at https://www.youtube.com/watch?v=_Bgp-twcGHw and at www.tinyurl.com/EbolaLocalABHR

An intervention coordinated in 2014 in Liberia by the Swiss government enabled the local production of alcohol-based hand rub (ABHR) solutions. ABHR can be defined as the first step of the IPC strategy, and have been shown effective on Ebola virus [26, 27]. The agreement and support of the Liberian authorities was obtained, as well as the validation of the WHO-ABHR formulation. In close collaboration with the Ministry of Health, three hospitals were selected: a mother and child institution, a major city hospital and a rural healthcare centre.

The intervention was devised in close collaboration with the University of Geneva Hospitals (HUG) and the WHO Collaborating Centre on Patient Safety at the University of Geneva Hospitals and Faculty of Medicine, which delivered ten ready-to-use kits to produce the WHO-ABHR. Each kit contained 1500 small plastic bottles, cans of glycerine and peroxide, an ethanol metre, laboratory equipment and some jerry cans. It was necessary to buy ethanol locally, because it cannot be shipped by air due to security regulations. In September 2014, it proved possible to find 220,000 l within Sierra Leone. A two-day training programme was set up and deployed over the initial three pilot hospitals and then a further seven hospitals, mainly in rural areas. Quality control and project evaluation were ensured.

A first group of twenty pharmacists was trained in November 2014, leading to each of the three hospitals producing over 1000 ABHR bottles. Strong motivation and considerable pride were observed in project participants. The staff of the participating hospitals was informed; monitoring showed that over 90 % of staff in each institution were aware of the project.

It is clear that measures enabling healthcare centres and populations to produce ABHR locally will be useful beyond the epidemic. It is therefore important to ascertain what capacity is already present to help create such solutions. In Guinea, there is currently no industrial production capacity for ethanol. In order to achieve production in this country, an option may be to revive the local sugar cane distillation industry, by opening a distillery or reopening one that was closed a few years before. Such a development would increase the autonomy and long-term sustainability of this project.

**Fig. 2:**
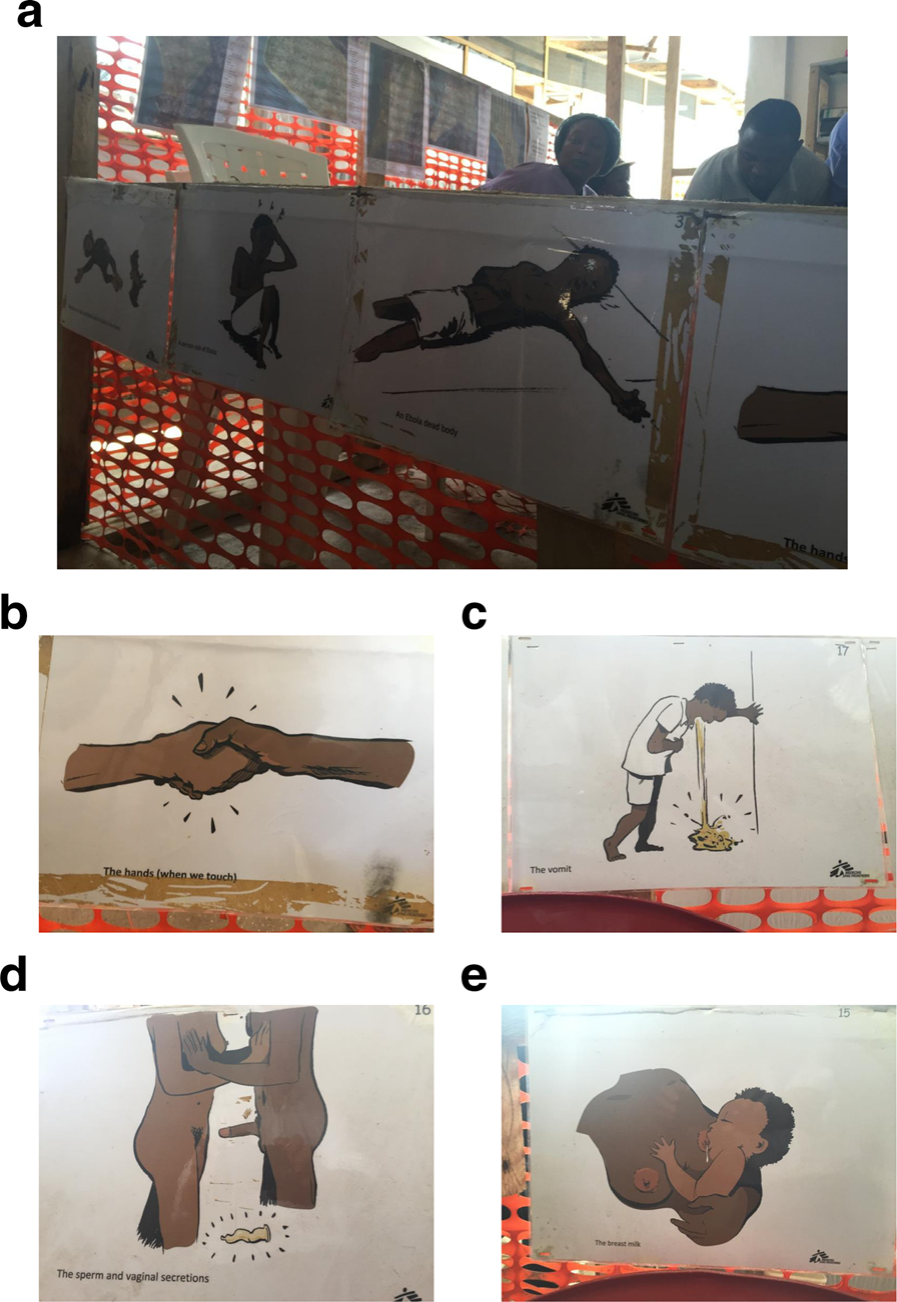
**a** Ebola treatment centre (ETC.), Redemption Hospital, Monrovia, Liberia. Waiting room in triage area with basic infection prevention measures for patients, relative and visitors (more details in **b** to **e**) (photo, D. Pittet). **b** to **e** Ebola treatment centre (ETC.), Redemption Hospital, Monrovia, Liberia. Waiting room in triage area with basic infection prevention measures for patients, relatives and visitors illustrating possible modes of transmission of the Ebola virus, through direct contact (**b**), and body fluid exposure (ie. such as vomit) (**c**), sexual intercourse (**d**), and breast feeding (**e**) (photo, D. Pittet)

Follow-up missions conducted in Liberia and in Guinea in October/November 2015 confirmed the sustainability of the project regarding the local production of ABHR, including quality control, and called for the necessity to train locals in IPC (www.tinyurl.com/EbolaLocalABHR). Adaptation of infection prevention education approach is illustrated in Fig. 2a to e). Furthermore, there was no nurse and no physician trained in IPC in Liberia before the EVD outbreak. As part of the Swiss-supported project, a Liberian nurse received a specific training in IPC between June and October 2015, and is currently setting up a curriculum for IPC in Liberia with support of the Ministry of Health, which has set up a national task force for IPC.

## Community engagement

Video at https://www.youtube.com/watch?v=UXjlJKQ5qtw

The response to the EVD epidemic demonstrated the need to improve the cultural congruence of interventions and to empower the community; those are critical for success. Disease outbreak and control require a co-created environment of cultural humility, reciprocal trust and respect, co-learning, community empowering action.

Researchers based in South Africa developed the CARE model, with practice-based evidence and key steps. Such a protocol aims at maintaining culture while describing risks and dispelling myths. It aims at avoiding bodily contact while ensuring sufficient time and space for praying, singing and/or dancing when the body of a family member is taken away.

In the context of the 2014–2015 EVD epidemic, the CARE framework was used to promote IPC community-engaged measures. Interventions were branded responses rather than interventions, in order to emphasise that the community is part of the solution. Based on practice-based evidence [28], the approach draws on participatory approaches. The key steps are indicated in Table 3. Most importantly, a process of community assets mapping creates an inventory of capacities rather than a bundle of deficits or pathologies. The community must be able to ensure prevention, treatment and self-care in collaboration with local government. This approach requires field testing in various settings. However, the flexibility and reflexivity of the framework would make it potentially applicable in other settings.

**Table 3:** Promoting infection prevention community-engaged measures against Ebola virus disease, 8 key steps (CARE framework)

-	Prepare to enter the affected community with a respected local leader
-	Enter the community with cultural humility and critical self-reflection about one’s own biases and beliefs
-	Identify key male and female community leaders who are influential in decision making
-	Empower the community leaders
-	Organise regular meetings where medical teams can be invited as participants rather than conveners
-	Assess to what extent communities are ready for change (knowledge of the issue and of the efforts involved in the response, leadership attitude)
-	Map the various groups that have resources and insights into the situation (religious groups, children, etc.)
-	Plan for sustainability, i.e. after the departure of the medical team. Most importantly, a process

## A role for anthropologists

Video at https://www.youtube.com/watch?v=-ml02V6biT0

A role for anthropologists emerged during the EVD epidemic because of a context of community resistance against physicians, especially in Guinea. Frequent physical contact with deceased people, surrounded by myths as well as social, religious, political and cultural conflicts, and confounded by the influence of traditional healers, all had negative consequences in the context of this epidemic. There were several reports of vandalism, demonstrations and even physical violence; this led to violent deaths of HCWs in Guinea during the course of the EVD crisis.

The objective of anthropologists in the epidemic response was to understand why and how resistance to HCWs developed. They deployed a multimodal strategy based on sound knowledge of local history and customs and appropriate choice of entry persons to observe social practices and discuss with communities. The objective was to find common grounds between the needs of the community and healthcare priorities.

In April 2014, a first group of healthcare experts was deployed with an anthropologist in an area where MSF had just installed an ETC, in a context of almost 100 % mortality. There were rumours that MSF was killing people and an anti-MSF demonstration had been planned. Anthropological activities with communities used community leaders to sensitise the population. Then, central government local representatives organised a large public meeting. As a result, the anti-MSF demonstration was cancelled.

In conclusion, community resistance can represent a considerable challenge in the context of emergency responses to disease outbreaks such as EVD. In such a context, anthropological interventions represent an emerging good practice and can bring significant results in terms of enabling the response against the disease, protecting HCWs and achieving buy-in from local communities.

## Diagnosis and treatment

Video at https://www.youtube.com/watch?v=7HjC8UtzVbY

Diagnostic issues are affected by biosafety regulations that need to be followed when investigating viruses such as the Ebola virus. When a patient presents with clinical symptoms, he usually already has a high viral load [9, 29]. Cultures are not performed in clinical routine for such viruses. Reference segments (conserved targets) in the viral genome are needed for PCR-based diagnosis, although it may be necessary to adapt assays to the genetic drift of the virus. Some assays were used both in the field and in high resource settings during the epidemic in West Africa [10, 30], despite the lack of detailed clinical validations in scientific publications.

Among lessons learned during this epidemic, the fact that primers and probes for RT-PCR are not being shared is a problem that needs to be solved at international level. Further issues were dicted by the fact that viral kinetics are not fully understood. It is accepted that viral load starts to decrease around day 6 after onset of disease, somewhat earlier in survivors than in non-survivors [29]. Moreover, RT-PCR accuracy is limited by limits of detection and quantification, and it is not uncommon to obtain both positive and negative results in the same sample when viral loads reach low levels [30].

During the 2014–2015 outbreak, it has been common to discharge patients as soon as blood sample proved negative by RT-PCR [31]. However, Ebola virus can persist in immunologically protected body compartments [32–34]. Among the various assays on the market, so-called loop-mediated isothermal amplification assays may prove interesting in the future [35]. Rapid point-of-care tests, are based either on genetic (PCR) or immunographic methods. The ReBOV antigen rapid test has been cleared for emergency use by WHO and the US Federal Drug Administration (FDA) in February 2015 [36]. However, its overall performance (sensitivity 92 %, specificity 85 %) may be a challenge in settings with high disease prevalence.

Direct acting antiretroviral therapies emerged with ZMAb, a cocktail of three monoclonal antibodies, shown to be efficient against Ebola virus in non-human primates [37]. ZMAb was not designed for clinical settings and is still in an experimental stage although it was delivered to at least 6 patients in the context of the current epidemic [30, 38]. Zmapp, a combination of chimeric antibodies targeting three different epitopes on Ebola virus which proved 100 % protective in guinea pigs and non-human primates inoculated with the Kikwit variant of the virus [39]. Animal trials have been conducted using the Makona variant with similar results regarding efficacy, despite there being at least 26 non-synonymous mutations on epitopes recognised by the monoclonal antibodies in the drug [40]. Indeed, the Ebola virus is evolving and mutating constantly. The concept of Zmapp is that by targeting several epitopes at the same time the chance of the drug being efficacious in humans will be increased. MIL-77, manufactured by a Chinese company, is another cocktail of humanized monoclonal antibodies produced in Chinese hamster ovary cells. It has been used as post-exposure prophylaxis agent in patients presenting with high-exposure risk [41]. There is currently no published information in the international literature that demonstrates the efficacy of MIL-77 in animal models or in humans.

Favipiravir, approved in Japan against influenza, is a broad-spectrum antiviral pyrazinamide derivative that functions as a viral RNA polymerase inhibitor [42, 43]. Preliminary results from a non-randomised clinical study in Guinea showed a reduction in mortality among treated patients, and especially among those without renal failure on admission and with lower viral load [44].

TKM Ebola is a cocktail of small interfering RNAs that target various viral proteins. It is efficacious in protecting Rhesus monkeys [45, 46]. It has been used in a few patients [47] and a clinical trial has been conducted in Sierra Leone. Plasma from convalescent individuals has been shown to be protective in non-human primates, and in a few humans [48]. There has been compassionate use of this approach during the current epidemic [47, 49, 50] but first results of a large-scale clinical trial showed no benefit in survival [51]. Brincidofovir was studied in a clinical trial in Liberia that had to be stopped due to the decline in the total number of cases. It is efficacious against DNA viruses; [52] its activity against RNA viruses is unknown, and no further clinical trial is planned at the present time. Synthetic antisense oligonucleotide analogues (resistant to RNase) called PMOplus have also been preven effective in animal models [53, 54]. There are around 50 more examples of experimental drugs not used in clinical practice at this time. For example, in Foya in 2014, MSF found that artesunate-amodiaquine reduced mortality [55].

In summary, perhaps as many as 50 drugs are currently in the research and development pipeline; there is however no current validated treatment. Pilot studies are needed. They should be randomised controlled trials wherever possible, but other trial setups may be necessary because randomisation is not always feasible, ethical, nor fast enough.

## Ebola and HIV

Video at https://www.youtube.com/watch?v=2o7eErripP4

At least 200,000 people are estimated to be living with HIV in Guinea, Liberia and Sierra Leone, one-quarter of whom are currently taking antiretroviral therapy. The continuity of HIV prevention and care was an important issue during the 2014–2015 EVD epidemic. Individuals were reluctant to attend any medical facility due to fear of Ebola infection and mistrust of medical services. Visits to HIV facilities in Conakry, the capital of Guinea, plummeted: the proportion of people not going to their HIV visit, and who had still not gone to one 90 days later, went from zero to 42 % between April and December 2014 [56].

It is critical to maintain a minimum HIV service package during epidemics, including access to male and female condoms, safe blood transfusion services, access to antiretroviral therapy, prevention of mother-to-child transmission, TB/HIV services and post-exposure prophylaxis.

**Fig. 3:**
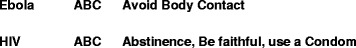
Similarities between Ebola and HIV prevention vocabulary

In terms of lessons learned, the EVD outbreak resonated strongly with HIV researchers and practitioners in West Africa and elsewhere because of similarities linked to the two viruses having a zoonotic origin in Africa and large societal impacts linked to stigma and discrimination. Other points perceived as being in common are the importance of surveillance, an elusive vaccine or cure, and challenges linked to procurement and logistics and the shortage of HCWs. It comes as no surprise that many HIV researchers and practitioners were among the volunteers working in ETC or clinical trial sites. Interestingly, an important aspect of the Ebola prevention vocabulary was ABC, for Avoid Body Contact. This resonates with the “Abstinence, Be faithful, use a Condom” used in the global response against HIV (Fig. 3).

Lessons from the HIV epidemic that could be of use when considering the EVD outbreak include: the importance of interventions to reduce stigma, the need for widespread screening programmes including point-of-care testing, engaging affected communities and ensuring continued access to treatment. Antiretroviral therapy adhesion adherence clubs – as developed by MSF in South Africa – have been used as an inspiration for ETCs in West Africa.

Another important issue is gathering evidence to improve care. Thanks to standardised monitoring forms introduced by MSF at the beginning of the EVD epidemic, it has been possible to obtain vitally important cohort data showing the effects of various antiretroviral treatments on EVD. These monitoring forms clearly do not correspond to an ideal study design. However, experience with HIV in the 1980s has shown that randomised placebo-controlled trials are not always possible.

## Ebola virus disease survivors

Video at https://www.youtube.com/watch?v=omImULFapeM

In June 2015, there were already more than 17,000 EVD survivors in West Africa. Sierra Leone had the highest number of survivors (around 4000) [57], followed by Liberia and Guinea. Despite growing numbers, little attention was being directed towards the continued treatment and rehabilitation of these persons with specific needs.

Although EVD was first described almost 40 years before the 2014–2015 outbreak, few articles concentrate on sequelae in survivors. A WHO report published in 1976 emphasised that “Those who did recovered did so slowly and painfully. Their appearance remained characteristic with deep-set eyes, drawn faces, a stooped walk and cachexia. Their condition slowly improved over several weeks, but many complained of pain and weakness for 6–8 weeks after discharge.” [58] Despite this knowledge, by June 2015, only few more studies could be found on the topic of EVD survivors [59–65], and only two of them are controlled studies [63, 66].

From March to June 2015, a team of the University of Geneva Hospitals (HUG) worked with MSF at the MSF survivor clinic of Freetown, to investigate the challenges faced by EVD survivors. A total of more than 160 survivors from the Western Area of Sierra Leone were followed there. Medical and mental health consultations were carried out, as well as prevention of STD and sexual transmission of EVD. Survivors were referred to the HIV national program to be tested. Local psychologists proposed personal counselling, family support and group discussions. Local support in the community was sought for in order to handle issues related to stigmatisation [67].

One important issue was that so-called “Certificates of discharge” were the only proof of survival status. These documents, issued first by the ETC. themselves then by government authorities, give access to free care and allow the bearer to work for an ETC. or in health promotion. However, they do not mean that the person is no longer infectious, and in practice the verification of such documents has proved difficult due to lack of communication between hospitals and to the presence of forged documents in the community.

In Sierra Leone, patients were discharged from ETCs when they had had one negative blood RT-PCR test, presented no acute symptoms and were considered autonomous in their movements. However, the Ebola virus is known to persist in various human body fluids. Virus has been detected in semen up to 284 days after onset of disease [33], and has been cultivated up to 82 days [34]. Aqueous humour of the eye has been tested positive for 10 weeks after onset of disease, in a patient presenting with severe uveitis, while no virus could be retrieved in the tears/conjunctival swab [64]. RT-PCR tests can detect the virus for days or even weeks after the last positive blood test in a variety of body fluids or tissues: sweat (24 days) [68], vaginal swab (21 days) [34], amniotic fluid (15 days) [69], breast milk (8 days) [32] and saliva (4 days) [34], urine (36 days) [70]. A nurse treated in the UK presented close to death with symptoms of advanced meningitis has been tested positive for Ebola virus in cerebro-spinal fluid 9 months after onset of disease [71]. There is no data in the literature on persistence of the virus in intra-articular fluid.

Two questions may be raised by these data. The first is that IPC issues remain vitally important after discharge from hospital. The second is that EVD may be considered as a sexually transmitted disease. Indeed, a case has been described of a woman infected by Ebola virus and whose only risk factor was a sexual activity with a survivor [59, 72].

Post-EVD complications include neuropsychological disorders (headache, irritability, memory loss, depression and other symptoms related to traumatic stress disorder), hair loss, hearing loss, thyroiditis, arthralgia/myalgia, anorexia, abdominal pain, myocarditis/pericarditis, tachycardia, skin desquamation and skin rashes [60–63, 65, 66, 73–77]. Ocular complications are encountered in up to 60 % of survivors, including sight-threatening uveitis, which have been shown to be more frequent in patients with higher viral load at admission [74].

Among the survivors followed in Sierra Leone [67], preliminary data showed that most frequent symptoms at first visit included suspected ocular complications (37 %), joint pain (39 %), headache (45 %), fatigue (27 %), anorexia (26 %), myalgia (24 %), insomnia (9 %) hair loss (10 %) or suspected cardiac complications (4 %). The most common ocular complaint was uveitis. Anterior uveitis and panuveitis were the most common subtypes of the disease. Among patients who attended a mental health consultation, a majority said they felt unhappy, nervous, tense and/or worried. In this respect, the lack of qualified psychologists is a major impediment for the continued treatment and rehabilitation of EVD survivors, in Sierra Leone and elsewhere.

In summary, EVD survivors suffer from physical complications compounded by a range of psychological and social problems and challenges. The physiopathology of the post-EVD syndrome remains unknown. Research is still needed to understand the full range of sequelae/complications and how the Ebola virus can persist in immunologically protected body fluids of survivors.

## Ebola patient treated in Geneva

Video at https://www.youtube.com/watch?v=upxyxopNKDc

The HUG started preparing for receiving a possible EVD patient in April 2014. Personnel from the intensive care and emergency departments were trained for EVD ahead of time (see video “Ebola PPE coaching” at http://tinyurl.com/EbolaPPEcoaching). Training and simulations were carried out long before it was known whether a patient might ever be admitted. Furthermore, the Swiss National Reference Centre for Emerging Viral Diseases, part of the Laboratory of Virology at HUG, was experienced in PCR-based investigation of viruses such as Ebola.

In August 2014, as the epidemic was growing in West Africa, HCWs were increasingly becoming infected. One month after the WHO call on 8 August 2014, specific training carried out within 3 weeks in Cuba enabled the first wave of 163 Cuban physicians to be deployed in West Africa. In November 2014, there were over 11,000 EVD cases declared, more than 5000 deaths, and the epidemic was spilling over into neighbouring countries.

On 18 November 2014, WHO called the Swiss Federal Office of Public Health to suggest the transfer of a patient with EVD. The transfer took place on 20 November. The patient was admitted to HUG on 21 November with high fever and swollen face features, and was in a semi-delirious state. Central and bladder catheters were already in place. Three hours after the patient’s arrival at the hospital, he received his first medication. We refer the reader to a complete description of the clinical and laboratory observation published elsewhere [30].

Between August 2014 and June 2015, a total of 27 EVD patients were treated in Europe or North America, among which 20 were evacuated – “medivaced” – after being infected in West Africa (the others declared the disease while already in Europe or North America). Weekly conference calls took place between the various centres treating the patients to exchange experiences and learn from each other.

Among lessons learned is that it is critical to be prepared ahead of time and that teamwork between disciplines is essential, in particular between virology, IPC, and emergency and intensive care medicine experts. Procedures need to already be in place for patient transfers to and within the hospital, practical arrangements including food and beverages, toilets and waste disposal. Laboratory testing including blood tests was carried out in patient’s room (see video “Ebola In Room Laboratory‬” ‬at http://tinyurl.com/EbolaInRoomLab) thanks to point-of-care devices, except for the RT-PCR, which was performed at Laboratory of Virology. It has been suggested that entry and exit points to a secure room should be different. At HUG, this was not the case because of architectural constraints; it was not considered a major problem, although it complicated waste management. Altogether, some 70 HCWs were involved in the team taking care of the single patient admitted at HUG.

Preparing for an EVD patient is a significant burden for any hospital that has to continue functioning while all events and procedures linked to EVD are taking place. In particular, advanced and well-trained IPC specialists are needed to develop recommendations and train personnel. Four sectors had to be prepared for the possible admission of an EVD patient: critical care, emergency wards for adults and children, as well as internal medicine. A one-hour practical training module was set up at HUG and was delivered to 250 HCWs. A total of 20 supervisors, mostly IPC staff, were trained and on duty for training supervision. Following trainings, staff had the possibility to repeat donning and doffing procedures on multiple occasions and permanent dedicated locations with PPE material were set up to allow individual or supervised training sessions to occur. Simulations and simulation sessions’ debriefings were also organized. Finally, a procedure was set out in case of an accidental exposure of a staff member to the virus.

After the arrival of the patient, three intensive care nurses worked in 3 × 8 h shifts. The team consisted of nine nurses: two in the room with the patient and three outside for supervision and preparedness. A so-called “buddy system” was put in place, as well as supervision by IPC team leaders. A total of 5 ICP nurses (3 in the morning; 3 in the afternoon; 1 at night) and at least 1 ICP senior physician (including at night) were necessary for continuous supervision and support of the staff entering and exiting the patient room (see Ebola training video recorded in real life while the patient was treated at HUG: “Ebola: entering and exiting the room” at https://youtu.be/PFbPL7_jEQY). Around 2500 nursing hours were spent with this single patient [78]. The final cleaning of the room after patient discharge requested a total of 15 HCWs who proceeded to the decontamination of the dedicated area and to the cleansing of the used materials, equipments and environment in one day.

Finally, sustained media attention is to be expected, with the risk of overpowering the hospital press offices and local health authorities. The HUG therefore contracted a team of science writers in advance, to issue timely and scientifically sound press releases in English and French throughout the treatment of this patient.

## Caring for Ebola patients in Washington, DC

Video at https://www.youtube.com/watch?v=9a26KVrWU38

There are similarities in preparation and planning among the units used for EVD patients in Europe and North America. The high containment unit at NIH near Washington DC, was one of the three hospitals in the USA (others were in Atlanta and in Nebraska) that was shortlisted to possibly receive a patient by the US federal authorities. The NIH had prepared from 2009 a safe containment room for patients with any infectious conditions [79]. It contains four rooms, one of which is a high-level containment isolation room. In addition, the unit is supported by a room with one large and two small autoclaves.

Table 4 lists the main elements identified as success factors for handling an EVD patient. When it was thought that a patient might actually be admitted, the atmosphere changed. An internal call for healthcare volunteers was highly successful and a flexible staffing model was developed. A “buddy system” was used, with two nurses in the room for a critically-ill patient, one in the anteroom and one at the nurses’ station. Trained observers were used and rapidly proved to be critical for ensuring the adequacy of donning and doffing procedures. Anticipated laboratory requirements were established. Daily debriefings established what worked and what could be improved. A clear, transparent communication plan was set up, with neighbouring institutions including other hospitals and schools.

**Table 4:** Caring for Ebola patients in the Northern hemisphere; main success factors

- Designating a single individual who is in charge	
- Team building with input from every team member	
- Institutional leadership	
- Developing efficient procedures and precautions	
- Developing and testing standard operating procedures (and modifying them as needed)	
- Using a “buddy system”	
- Training of observers for ensuring the adequacy of donning and doffing procedures	
- Anticipating on laboratory requirements	
- Daily debriefings (once the patient is admitted)	
- Clear and transparent communication	

One of the patients arrived at a different airport from the three with which the NIH had worked out procedures. Because the NIH had not been in contact with the mayor or council of the county involved, these activities had to be conducted as the patient was arriving. Another major issue was managing hysteria and anxiety, some of which was similar to what had occurred in the 1980s when HIV patients were admitted to the same hospital. Interestingly, the MSF and WHO declarations about the epidemic provoked less media interest than the arrival of EVD patients on American soil. Altogether, 12 patients were treated in North-America and ten of them survived. Being able to provide care for these patients in environments in which physiological and other studies could be conducted during the provision of care provides an opportunity to learn more about the pathogenesis and pathophysiology of the disease.

## Ebola preparedness in New York

Although there was no federal plan for New York to receive EVD patients, the city had procedures in place because of its strategic position as the main entry point to the USA. New York had an experience of media frenzy when, in October 2014, an American physician who had been working on Ebola in Liberia declared symptoms compatible with EVD. On 23 October 2014, he reported himself to public health authorities and was promptly transferred to Bellevue hospital. The following day, in neighbouring New Jersey, a nurse returning from Sierra Leone was confined to a quarantine tent for three days despite having no EVD-specific symptoms. Subsequent testing proved that she was virus-free. When she succeeded in freeing herself from that situation, authorities in her state of Maine also attempted to quarantine her.

The male patient, who identified himself as Dr Craig Spencer, subsequently wrote a critical position paper on the attitude of the media and of key political figures towards EVD [80]. In his view, the threat of a 21-day quarantine may cause sick people to conceal symptoms, defer seeking treatment, misreport their exposure or alter their travel plans to avoid quarantine. Also, Spencer underlined that allowing restrictions such as quarantines to occur when they are not in line with official public health recommendations (issued by the CDC for the USA) undermines and erodes confidence in the ability of citizens to respond cohesively to public health crises. Other critics believe that there was too much confidence that the USA and its institutions could handle EVD cases. There was a failure to understand how a fatal EVD case in Texas, which occurred on 8 October 2014, could ignite fear and many misconceptions across the country.

## Treatment in Sierra Leone and Geneva

Video at https://www.youtube.com/watch?v=hq_G1Rta2EU

A comparison of the handling of EVD patients in Switzerland and Sierra Leone by physicians who took care of such patients in both countries may seem speculative, but can be useful if the objective is to achieve health equity (Table 5). In the well-equipped isolation room in the intensive care unit at HUG, a large number of staff were available to treat a single patient (see above). A complete virology laboratory was at hand, with reverse transcription real-time PCR (real-time RT-PCR), ELISA assays for IgG and IgM, and haematological and chemical tests that could be performed at the bedside. The patient had access to two experimental treatments: the ZMAb cocktail containing three EBOV-glycoprotein-specific neutralising monoclonal antibodies and the antiviral favipiravir (see above). An important point is that a lot of personnel and time were necessary, to treat a single patient. Graphs were produced every day and sometimes several times a day, to monitor the dynamics of EVD and body responses. For example, it could be observed that the emergence of the IgG response in this patient took 11 days, with a rapid increase in the IgG titres thereafter [30].

**Table 5:** Similarities and differences regarding Ebola patient management at the University of Geneva Hospitals, Switzerland, and in a MSF Ebola Treatment Center in Freetown, Sierra Leone

	University of Geneva Hospitals	Freetown MSF ETC.
EVD diagnostics	Real-time RT-PCR (Altona kit): Daily viral load estimation, in plasma and other body fluids	Real-time RT-PCR (Altona kit): One for diagnosis, one for discharge
Point-of-care lab tests	Haematology Blood gases Basic coagulation assessment Biochemistry (including CRP, electrolytes, CK, renal function and liver tests) Malaria and Dengue RDTs	Biochemistry (including CRP, electrolytes, CK, renal function and liver tests) Malaria and pregnancy RDTs
Patient equipment and monitoring	Central venous line Urinary catheter Nasal oxygen delivery as needed Continuous temperature, oxymetry and BPM monitoring Regular BP measurements	Peripheral venous line (not all patients) Nasal oxygen delivery (2 devices for the whole ETC.) Temperature, BP, BPM and oxymetry measurements (twice daily)
Treatment	Patient-tailored Experimental treatments: ZMAb and favipiravir IV nutrition	Defined protocol including IV or oral rehydration, electrolytes, antibiotics, antimalarials, analgesics, antiemetics, spasmolytics and omeprazole

One of the responsible physicians at HUG also went to the Prince of Wales Ebola treatment centre (ETC.) in Freetown, Liberia. This MSF-run hospital consisted of 4 tents and could handle up to 100 patients. Many patients would arrive on foot at the triage area. Suspected cases were separated from each other and then, according to the RT-PCR result, were directed to the intensive care tent or to the oral tent, depending on the clinical phase of each patient.

Among the key differences between the two settings are the high number of trained staff who are available per patient in well-resourced countries. This is one of the reasons why clinics in Europe and North America were able to tailor treatments, whereas in Africa it was always necessary to follow protocols. Equipment was clearly more readily available in Geneva, although interestingly the same diagnostic RT-PCR kit was used in both locations. The main and most shocking difference was the case fatality rate: on the 27 cases treated in Europe and North America, it was 18.5 % in high-resource countries against around 50 % in West Africa.

For the future, it is necessary to define life-saving measures to be rolled out as a priority in low-resource settings. It is not known if the priority should be aggressive monitoring and supportive care, or if the limited resources should be concentrated on delivering antiretroviral therapy. Similarly, not enough is known about the pros and cons of using a central venous catheter, which is very useful for blood sampling and aggressive rehydration, but which implies risks associated with device use and concerns about staff safety.

## Vaccines against Ebola virus

Video at https://www.youtube.com/watch?v=0BjCsrApth4

When the Ebola crisis was recognised as an international emergency in August 2014, the exponential increase in the number of cases and the increasing numbers of HCWs becoming infected led many public health experts to think that the only answer to the epidemic might be a vaccine. These thoughts led to a race towards an Ebola vaccine.

At that time, only two Ebola vaccine candidates had been shown to be 100 % protective in non-human primates and were available according to good manufacturing practice. Both were vector vaccines expressing the envelope gene of the Ebola virus Zaire strain. The first was ChAd3-ZEBOV, a live non-replicating chimpanzee adenovirus, licenced by NIAID (USA) to the GSK company [81]. Preliminary trials in the UK, USA, Mali and Switzerland had shown the vaccine to be safe with no serious adverse effects (although flu-like symptoms were frequent) [82–84]. A relatively high dose of 2 × 10^11^ plaque-forming units (PFU) was required to induce antibody responses. Subsequent trials on lower doses (between 1 × 10^10^ and 5 × 10^10^ PFU) were associated with satisfactory T-cell responses but that were not sustained for more than a few weeks. Therefore, the ChAd3-ZEBOV vaccine has been recommended as a “prime-boost” vaccine requiring at least two doses and a large primary dose (2 × 10^11^) for phase II/III trials.

The other Ebola vaccine candidate, rVSV-ZEBOV, is a live replicating vaccine based on the vesicular stomatitis virus, whose replicating genes are maintained, and whose envelope gene is replaced by the EBOV-GP envelope gene [85]. This vaccine was licensed to NewLink Genetics (and subsequently to Merck) by the Public Health Agency of Canada that went on to donate 800 doses to WHO to accelerate its international evaluation. WHO rapidly set up the VEBCON consortium containing four trials – in Gabon, Germany, Kenya and Switzerland – with a total of 250 study volunteers to evaluate the safety profile and dose selection for this vaccine.

Each of these trials was a phase I placebo-controlled randomised clinical trial, which relied heavily on the generosity of volunteers [86]. For the trial in Switzerland, regulatory and ethical approval was obtained from three sources (HUG, Swiss federal authorities and WHO) within one month. In Geneva, volunteers were randomised to one of three groups: 10^7^ or 5 × 10^7^ PFU or placebo. After initial screening, each volunteer received a single dose and was monitored closely for the ensuing days and weeks. Each week, 15 subjects were screened, enrolled, immunised and followed every week.

However, on 9 December 2014, a safety-driven study hold occurred because around 20–25 % of vaccinees were experiencing joint pain, which was identified as dose-independent viral arthritis. Another unexpected event, affecting around 10 % of vaccinees, was maculopapular rash and vesicular dermatitis on hands and feet, which was found to be associated with CD4+ T-cell infiltration. Rare cases of viral vasculitis were also observed. The understanding at present is that the rVSV vector is responsible for vesicular dermatitis that occurs on infection with the wild-type virus, but that arthritis and vasculitis are an intrinsic property of the vaccine [87]. The trial in Geneva started again on 3 January 2015.

The first published results of the four VEBCON trials were based on 138 healthy adult volunteers who had all been followed for at least four weeks [86]. It could be seen that viremia increased very rapidly – usually within 24 h – among subjects who had received a dose of several million PFU (doses ranged from 300,000 PFU – for which viremia increased less – to 3–50 million PFU where viremia peaked after 1–3 days at several thousand RNA copies/ml). However, whatever the initial dose, the virus appeared to have been cleared from the blood by day 7. Although very few participants had no adverse events, almost all were mild or moderate (fever or feverish feeling, flu-like symptoms including myalgia and fatigue) and lasted no more than one or two days.

Both adverse events and antibody response were clearly associated with the dose of vaccine received [87]. This led to the selection of 2 × 10^7^ as the dose of choice for subsequent phase III trials in West Africa. Three phase III trials began in West Africa between February and April 2015. However, because of the (most fortunate) reduction in the number of EVD cases in West Africa, it was not possible to carry out all the clinical trials that were planned. However, in July 2015, *The Lancet* published the first results from a phase 3 cluster-randomised trial of the rVSV-ZEBOV Ebola virus vaccine in Guinea including more than 7,500 indiviuals [88]. The interim analysis indicates that the vaccine might be highly efficacious and safe.

At the end of 2015, two candidate vaccines (single-dose rVSV-ZEBOV and prime/boost ChAd3) have entered phase II/III clinical trials, three other candidates are in phase I trials (ChAd3/prime boost), Ad26/prime boost, EBOV virus-like particles with glycoprotein) and the pipeline which was empty only months before is now full of preclinical candidate vaccines. The 18 months between mid-2014 and the end of 2015 have seen an unprecedented race against the clock to deliver a safe and efficacious vaccine against the Ebola virus. It is vitally important to pursue current efforts to develop such vaccines, because it would not be tolerable to face another EVD epidemic without having a safe and efficacious vaccine to offer at the very least to frontline workers.

## Conclusion

This overview concludes that lessons learned from the 2014–2015 EVD epidemic must be used to prepare for the next outbreak. Among the main lessons learned are that preparedness needs to be improved globally, health systems need to be strengthened, especially in rural areas, and interdisciplinary teams need to be constituted or at least planned for ahead of time. This epidemic also raised the critical question of the reforms needed at a global level for outbreak prevention and response. The report of the Harvard Global Health Institute and London School of Hygiene & Tropical Medicine Independent Panel on the Global Response to Ebola recently published in The Lancet suggests 10 essential reforms to conduct before the next pandemic [89]. These recommendations require high-level leadership political commitment for the proposed roadmap to be translated into concrete actions.

Emerging approaches such as MOOCs and the use of anthropologists embedded in medical teams have been a hallmark of the response to this epidemic. In order to understand and empower local communities in sensitive areas such as safe burials, there is a strong need for culturally sensitive approaches which can best be addressed by social science teams and medical teams working together, or better with teams including experts from various fields [89].

In this respect, a lot can be learned from the global response to HIV. The Ebola crisis saw a remarkable mobilisation of HIV researchers and activists. At short notice, information about the unfolding EVD epidemic was presented during HIV conferences. HIV levels were in the range of 1–2 % of the total population in the 3 countries most affected by the Ebola virus.

As of December 2015, this epidemic has claimed more than 11,000 lives and generated more than 17,000 EVD survivors. Too little is known about the pathogenesis and the treatment of EVD, so basic and applied research in this field is urgently required. Data on viral kinetics are still scarce, but viral load is clearly related to survival. Specific clinical features of the infection with the Makona variant may have led to extend the spread of the outbreak. Moreover, it is clear now that the virus can persist in immunologically protected body sites up to 9 months after an individual has been declared cured. This explains some of the complications and also poses several questions regarding sexual transmission of the disease.

The continued treatment and rehabilitation of the EVD survivors – who have faced near-death experiences and some of whom are orphans – is a public health priority. They commonly experience a “post-Ebola syndrome”, mainly consisting in debilitating joint pain, uveitis that can lead to blindness if left untreated, and psychological issues. The three countries most affected by the Ebola epidemic have almost no psychologists with the training necessary to face such a task. And little is known about how communities can cope with rumours and fear related to tragic and disruptive events such as an EVD epidemic. Such problems need to be addressed within the general framework of health systems recovery and development, and require integrated medical and social science approaches.

New cases of Ebola have been confirmed in Guinea and Liberia just after West Africa has been declared "Ebola free", and more flare-ups are likely to occur, due to delayed virus clearance in the survivor popoulation. One has to highlight the unprecedented commitment of the international community in developing an effective vaccine against this dreadful threat.
